# Early-Onset Sepsis in Preterm Neonates of 22-28 Weeks’ Gestation: An International Cohort Study

**DOI:** 10.1016/j.jpedcp.2026.200216

**Published:** 2026-05-14

**Authors:** Husharn L. Duggan, Prakesh S. Shah, Malcolm Battin, Gil Klinger, Mark Adams, Maximo Vento, Vieri Lastrucci, Ligia Maria Suppo Sousa Rugolo, Maria Regina, Stellan Håkansson, Tetsuya Isayama, Mikael Norman, Satoshi Kusuda, Brian Reichman, Liisa Lehtonen, Kjell Helenius, Kei Lui Kei, Kenneth Tan, iNeo Investigators

**Affiliations:** 1 Department of Paediatrics, Monash University, Clayton, Victoria, Australia; 2 Department of Pediatrics, University of Toronto, Toronto, Canada; 3 Newborn Services, Auckland City Hospital, Grafton, Auckland, New Zealand; 4 Department of Neonatal Intensive Care, Schneider Children's Medical Center of Israel, Petah Tikva, Israel; 5 Department of Neonatology, University and University Hospital Zurich, Zurich, Switzerland; 6 Division of Neonatology, Health Research Institute Hospital La Fe, Valencia, Spain; 7 Epidemiology Unit, Meyer Children's IRCCS, Florence, Italy; 8 Division of Neonatology, Botucatu Medical School, São Paulo State University (UNESP), Botucatu, Brazil; 9 Section of Pediatrics, Department of Clinical Sciences, Umeå University, Umeå, Sweden; 10 Division of Neonatology, National Center for Child Health and Development, Tokyo, Japan; 11 Department of Neonatal Medicine, Karolinska University Hospital, Stockholm, Sweden; 12 Division of Pediatrics, Department of Clinical Science, Intervention and Technology, Karolinska Institutet, Stockholm, Sweden; 13 Neonatal Research Network of Japan, Kyorin University, Tokyo, Japan; 14 Gertner Institute for Epidemiology and Health Policy Research, Sheba Medical Centre, Tel Hashomer, Israel; 15 Department of Pediatrics and Adolescent Medicine, Turku University Hospital, Turku, Finland; 16 National Perinatal Epidemiology and Statistics Unit, University of New South Wales, Randwick, Australia

**Keywords:** epidemiology, extremely preterm infants, international registry, neonatal sepsis

## Abstract

**Objective:**

Early-onset sepsis (EOS) is associated with increased mortality and morbidity in preterm neonates. We aim to study the epidemiology of EOS among extremely preterm neonates across a multinational neonatal registry.

**Study design:**

A retrospective cohort study (2007-2023) of neonates born under 28 weeks’ gestation from the International Network for Evaluation of Outcomes in Neonates collaboration, involving 11 national neonatal registries. The definition of EOS was blood or cerebrospinal fluid culture-positive sepsis within the first days after birth (ranging from <2 to <7 days of life). Primary outcome was mortality. Secondary outcome was major morbidities (severe neurological injury, severe retinopathy of prematurity, necrotizing enterocolitis, late-onset sepsis, or bronchopulmonary dysplasia). Univariate and multivariate analyses were performed.

**Results:**

Four thousand three hundred fifty-four of 130,938 (3.3%) neonates with mean (±SD) gestational age 26.1 (±1.7) weeks and birthweight 874 (±241) grams developed EOS. Rates of EOS ranged from 1.6% to 6.6% across International Network for Evaluation of Outcomes in Neonates countries. Pregnancy associated hypertension and caesarean delivery were not associated with EOS. For EOS infants the mortality rate was 35.5% and mortality or morbidity rate was 78.2%. Odds of mortality remained higher after adjustment (AOR, 2.49; 95% CI: 2.27, 2.74) along with mortality or morbidity (AOR, 1.89; 95% CI, 1.70-2.10). Severe neurological injury was the leading complications (AOR, 2.34; 95% CI, 2.11-2.59). Variations in incidence, morbidity, and mortality were also noted between countries.

**Conclusion:**

EOS is common in extremely preterm neonates, varying across countries and associated with higher odds of mortality and morbidity.

Early-onset sepsis (EOS) remains a serious global issue, contributing to increased neonatal mortality and morbidity worldwide, particularly in low and middle-income countries.^1^ Nevertheless, EOS also remains an important public health issue in high income countries. In particular, being born extremely preterm (EP) and of very low birth weight have been recognized as significant contributors to EOS incidence and mortality.^2^, ^3^, ^4^, ^5^, ^6^ and has become a focus of research in recent years. Recent studies have highlighted variances in microorganism profiles, risk factors, and outcomes between term and EP neonates,^7^ therefore demonstrating the need for specific research into this cohort.^2^ However, current studies remain limited geographically and it is difficult to translate these findings to the worldwide context due to notable variances in neonatal care and outcomes across different countries.^8^ Giannoni et al. identified this issue when comparing late preterm and term EOS among 11 countries across North America, Europe, and Australia, describing wide variations in incidence, mortality, and antibiotic exposure.^9^

The International Network for Evaluation of Outcomes in Neonates (iNeo) is a collaboration of 11 multinational neonatal networks comprising of 10 high-income countries and 1 middle-income country, utilizing standardized data to highlight gaps in care that can inform improvements in health policies among tertiary neonatal intensive care units (NICUs).^10^^,^^11^ Previous studies have analyzed EOS in preterm neonates using data from individual participating neonatal networks of iNeo,^3^^,^^12^ and a two-network comparison,^4^ but a comprehensive study across multiple networks has not been done. This study aims to expand current literature and provide a standardized comparison of EOS incidence, risk factors and outcomes among neonates of 22-28 weeks’ gestation utilizing the multinational database, iNeo. However, this paper does not make comment on the causative microorganism profiles of EOS among each country.

## Methods

### Study Design

This retrospective cohort study utilized collected data of neonates born at 22^0^-28^6^ weeks’ gestation, admitted to a participating unit of the iNeo database between 1 January 2007 and 31 December 2023. Neonates >1499g were excluded from the study, as a birth weight cut-off of <1500g was a registration requirement of many of the participating networks. Neonates diagnosed with a major congenital anomaly were excluded. Neonates who were transferred between participating units were only counted once and those admitted for the first time after 36 weeks postmenstrual age were excluded. As data of stillborn deliveries and delivery room deaths were not routinely collected by all units, these were not included. The differences between neonatal networks for population coverage, data collection and included units are described in the Supplementary Table.

### Ethics and Data Management

Each participating network holds individual research ethics approvals for collection of data within each country. Most networks either extract data directly from patient records or participating units enter data directly into a central database, with the exception of certain networks.^8^ All data were transferred to and handled by the iNeo Coordinating Centre at the Maternal-Infant Care Research Centre, Mount Sinai Hospital, Toronto, Canada. The iNeo Coordinating Centre also obtained research ethics board approval for data transfer and all research projects facilitated by the iNeo collaboration.

### Covariates

Gestational age (GA) was estimated in hierarchical order of early ultrasound, timing of last menstruation, obstetric estimate, and physical examination after birth. Birth weight z-scores were calculated utilizing birth weight references from each country. Data on antenatal corticosteroid administration, hypertension in pregnancy, delivery of twins or of higher order, and mode of delivery were recorded. (See Table 1).

**Table 1:** Characteristics of EOS across the iNeo collaboration

Characteristic	Total population	EOS	No EOS	*P* Value
Antenatal corticosteroids, N/Total N (%)	102936/127096 (81.0)	3369/4286 (78.6)	99567/122810 (81.1)	<.001
Hypertension in pregnancy, N/Total N (%)∗	18435/120779 (15.3)	402/4052 (9.9)	18033/116727 (15.5)	<.001
Male, N (%)	70153/130830 (53.6)	2323/4351 (53.4)	67830/126479 (53.6)	.76
Gestational age (weeks), mean (SD)	26.1 (1.6)	25.5 (1.7)	26.1 (1.7)	<.001
Birth weight (grams), mean (SD)	873.6 (241.0)	529.9 (236.1)	875.1 (241.0)	<.001
Birth weight z-score, mean (SD)	−0.1 (0.8)	0.1 (0.8)	−0.1 (0.8)	<.001
Caesarean birth, N/Total N (%)	84254/129615 (65.0)	2570/4348 (59.1)	81684/125267 (65.2)	<.001
Twins or higher order, N/Total N (%)	34627/130914 (26.5)	908/4354 (20.9)	33719/126560 (26.6)	<.001
Outborn, N/Total N (%)	11581/129034 (9.0)	262/4233 (6.2)	11319/124801 (9.1)	<.001
Mortality rate, N/Total N (%)		1545/4353 (35.5)	21117/126510 (16.7)	
Age of death (days), median (IQR)		5 (2-13)	7 (2-20)	

∗ No data for SNN 2007-15.Abbreviation: EOS (early-onset sepsis).

### Exposure and Outcomes

The primary exposure, EOS, was defined as blood or cerebrospinal fluid culture-confirmed sepsis within the first three days after birth. For Japan (NRNJ), EOS was considered until the first seven days after birth and for Australia and New Zealand (ANZNN), until 2 days after birth due to predefined coding criteria. As data on causative organism were not available from every network, EOS was reported as a binary outcome of EOS or no EOS. Polymicrobial infections were only reported once per neonate. The primary outcome was mortality and was determined by death prior to discharge or transfer to a step-down unit. Major neonatal morbidity was a secondary outcome defined by the presence of one or more of the following conditions: severe neurological injury, which included stage 3 or 4 intraventricular hemorrhage (IVH) with ventricular dilatation, as per the criteria by Papile et al,^13^ or parenchymal injury (including periventricular leukomalacia (PVL)) with or without IVH; severe retinopathy of prematurity, defined as either stage 3 or 4 according to the International Classification^14^ or the need for laser surgery or intraocular injections of anti-vascular endothelial growth factor; stage 2 or 3 necrotizing enterocolitis according to Bell's criteria^15^; culture-proven late-onset sepsis; and bronchopulmonary dysplasia defined as oxygen requirement at 36 weeks postmenstrual age or at transfer to level 2 units if transferred earlier. These major neonatal morbidities were also analyzed individually.

### Statistical Analysis

Incidence rates were determined by number of cases as a percentage of EP admissions to participating NICUs. Adjusted annual EOS rates are shown in Table 2. Standard descriptive analyses were used to determine differences between those diagnosed with EOS compared with those who were not for general characteristics across the entire cohort. Multivariable logistic regression analyses were used to determine adjusted odds ratios (AORs) for mortality and morbidity after controlling for GA, birth weight z-scores, sex, multiple births, and outborn birth, removing potential confounding factors that commonly contribute to mortality and morbidity in preterm neonates as well as which are not affected by practice variations between countries, for example, mode of delivery. Given the range of countries and ethnicities included in the study, it was expected that there would be differences in birth weights between networks. Therefore, country-specific birth weight z-scores were used for the multivariable logistic regression analyses over birth weights to facilitate standardization. Standardized ratios for comparative analyses between countries were calculated using an indirect standardization approach. The expected numbers of neonates with outcomes for each individual country were calculated from the multivariable logistic regression model constructed on the rest of the dataset. We applied Bonferroni correction for pairwise comparisons across countries. Standardized ratio estimates and the 95% CI for each individual country were displayed graphically. All analyses were 2-sided and conducted using SAS V.9.4 (SAS Institute), R V.4.4.0 (R Core Team) and Graphpad Prism V.10.2.0 (Graphpad Software).

**Table 2:** Early-onset sepsis incidence rate by year

Year	EOS rate	Adjusted EOS rate (95% CI)
2007	227/7165 (3.2)	(ref)
2008	260/7602 (3.4)	1.1 (0.9-1.3)
2009	269/7759 (3.5)	1.1 (0.9-1.3)
2010	294/8043 (3.3)	1.1 (1.00-1.4)
2011	276/8347 (3.3)	1.0 (0.9-1.2)
2012	263/8576 (3.1)	1.00 (0.8-1.1)
2013	284/8198 (3.5)	1.1 (0.9-1.3)
2014	302/9078 (3.3)	1.0 (0.9-1.2)
2015	331/8952 (3.7)	1.1 (1.00-1.4)
2016	314/8886 (3.5)	1.1 (0.9-1.3)
2017	293/8375 (3.5)	1.1 (0.9-1.3)
2018	292/8194 (3.6)	0.9 (0.8-1.1)
2019	234/7788 (3.0)	0.8 (0.6-0.9)
2020	209/7352 (2.8)	0.9 (0.7-1.0)
2021	220/7242 (3.0)	0.9 (0.8-1.1)
2022	225/7292 (3.1)	0.9 (0.8-1.1)
2023	61/2089 (2.9)	0.9 (0.6-1.1)
Total	4354/130938 (3.3)	

## Results

Between 2007 and 2023, a total of 137,366 neonates born between 22 and 28 weeks’ gestation and admitted to a NICU were identified among participating networks of the iNeo database. The Canada (CNN) network provided data between 2007 and 2023. The Australia and New Zealand (ANZNN), Finland (FinMBR), Israel (INN), Japan (NRNJ), Spain (SEN1500), Switzerland (SNN) and Sweden (SNQ) networks provided data between 2007 and 2022. The Brazil (BNN) Network provided data between 2014 and 2023. The Tuscany, Italy (TuscanNN) network provided data between 2009 and 2022. Neonates born >1500 grams (*N* = 1026) or with major congenital anomalies (*N* = 5402) were excluded, providing a final study population of 130 938 neonates. Table 3 outlines the frequencies of neonatal admissions, study populations, and rates of EOS within each network.

**Table 3:** Characteristics of each participating registry in the iNeo collaboration

Neonatal registry∗	ANZNN	BNN	CNN	FinMBR	INN	NRNJ	SEN1500	SNN	SNQ	TuscanNN	iNeo
Year	2007-2022	2014-2023	2007-2023	2007-2022	2007-2022	2007-2022	2007-2022	2007-2022	2007-2022	2009-2022	2007-2022
Gestational age 22-28 wks, N	24917	8037	27253	2865	9864	33730	17130	5047	7105	1418	137366
Birth weight >1499 grams, N	305	51	369	36	5	34	92	42	77	15	1026
Major congenital anomalies, N	1007	24	1142	102	156	1598	580	129	123	24	5402
Study population, N	23605	7445	25742	2727	9703	32098	16458	4576	6905	1379	130938
Early-onset sepsis N/Total N (%)	512/23605 (2.2)	192/7445 (2.6)	560/25742 (2.2)	44/2727 (1.6)	347/9703 (3.6)	1235/32098 (3.9)	1092/16458 (6.6)	177/4876 (3.6)	142/6905 (2.1)	53/1379 (3.8)	4354/130938 (3.3)
Antenatal corticosteroids, N/Total N (%)	21355/23605 (91.3)	4973/6523 (76.2)	22341/25235 (88.5)	2519/2678 (94.1)	7362/9693 (76.0)	18688/30580 (61.1)	14168/16266 (87.1)	4464/4840 (92.2)	5891/6521 (90.3)	1175/1371 (85.7)	102936/127096 (81.0)
Hypertension in pregnancy, N/Total N (%)†	3324/23598 (14.1)	2197/7405 (29.7)	3996/24992 (16.0)	166/2280 (7.3)	995/9674 (10.3)	4187/30639 (13.7)	2349/13988 (16.8)	396/2209 (17.9)	636/4620 (13.8)	189/1374 (13.8)	18435/120779 (11.9)
Male, N (%)	12722/23596 (53.9)	3902/7442 (52.4)	13878/25707 (54.0)	1435/2727 (52.6)	5351/9703 (55.2)	17138/32076 (53.4)	8623/16422 (52.5)	2605/4874 (53.5)	3765/6904 (54.5)	734/1379 (53.2)	70153/130830 (53.6)
Gestational age (weeks), mean (SD)	26.2 (1.5)	26.0 (1.8)	26.1 (1.6)	26.0 (1.8)	26.1 (1.7)	25.8 (1.8)	26.3 (1.5)	26.3 (1.4)	25.9 (1.8)	26.0 (1.7)	26.1 (1.7)
Birth weight (grams), mean (SD)	913.7 (236.2)	831.2 (242.4)	905.7 (238.2)	878.0 (252.7)	878.7 (235.7)	816.4 (239.9)	893.8 (226.8)	876.1 (232.3)	876.7 (251.3)	837.1 (255.9)	873.6 (241.0)
Birth weight z-score, mean (SD)	0.1 (0.8)	−0.2 (0.9)	0.1 (0.8)	0.1 (0.9)	−0.1 (0.79)	−0.2 (0.8)	−0.1 (0.8)	−0.2 (0.8)	0.1 (0.8)	−0.2 (0.9)	−0.1 (0.8)
Caesarean birth, N/Total N (%)	14012/23523 (59.6)	3955/7442 (53.1)	15281/25628 (59.6)	1636/2659 (61.5)	6376/9701 (65.7)	23788/31088 (76.5)	10111/16457 (61.4)	3932/4874 (80.7)	3932/4874 (80.7)	824/1379 (59.8)	84254/129615 (65.0)
Twins or higher order, N/Total N (%)	6161/23597 (26.1)	1637/7445 (22.0)	6636/25732 (25.8)	731/2727 (26.8)	3570/9703 (36.8)	6076/32098 (18.9)	6250/16457 (38.0)	1410/4876 (28.9)	1742/6905 (25.2)	414/1374 (30.1)	34627/130914 (26.5)

∗ Neonatal registries: ANZNN (Australian and New Zealand Neonatal Network), BNN (Brazilian Network on Neonatal Research), CNN (Canadian Neonatal Network), FinMBR (Finnish Medical Birth Register), INN (Israel Neonatal Network), NRNJ (Neonatal Research Network of Japan), SEN1500 (Spanish Neonatal Network), SNN (Swiss Neonatal Network), SNQ (Swedish Neonatal Quality Register), TuscanNN (Tuscany Neonatal Network), iNeo (International Network for Evaluation of Outcomes in Neonates).

† No data for SNN 2007-15.

### Early-Onset Sepsis and Incidence

**Figure 1:**
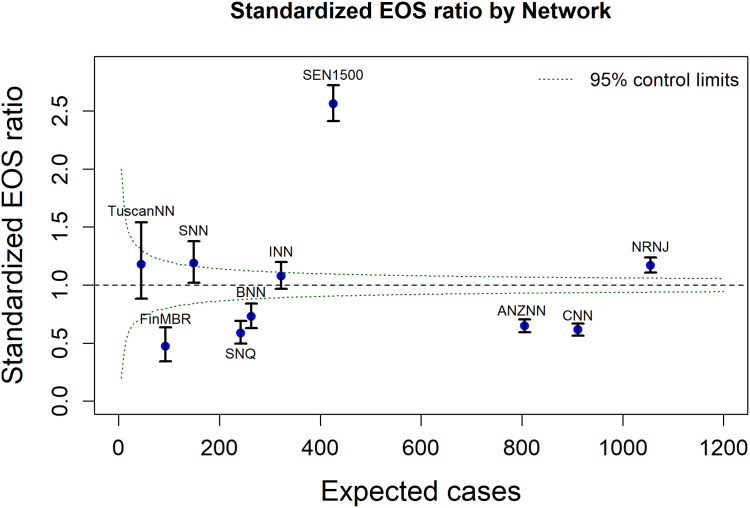
Comparison of standarized EOS ratio with expected cases by network. Points represent standardized ratios for comparative analyses between networks using an indirect standardization approach. Error bars represent 95% confidence intervals of standardized ratios for each network and the dotted lines represent the 95% confidence intervals of standardized ratios based on expected cases. *ANZNN*, Australian and New Zealand Neonatal Network; *BNN*, Brazilian Network on Neonatal Research; *CNN*, Canadian Neonatal Network; FinMBR, Finnish Medical Birth Register; *INN*, Israel Neonatal Network; *NRNJ*, Neonatal Research Network of Japan; *SEN1500*, Spanish Neonatal Network; *SNN*, Swiss Neonatal Network; *SNQ*, Swedish Neonatal Quality Register; *TuscanNN*, Tuscany Neonatal Network.

The total incidence of EOS across the iNeo collaboration was 3.3% with variability in incidence from 1.6% in Finland to 6.6% in Spain. Neonates diagnosed with EOS were more likely to receive antenatal corticosteroids and to be of lower GA and lower birth weight. Neonates diagnosed with EOS were less likely to have maternal hypertension in pregnancy, be delivered by caesarean section, be of twins or higher order, or be born outside of a hospital (Table 1). The incidence of EOS between 2007 and 2023 remained stable over time (Table 2). A comparison of standardized EOS OR with expected cases by network is graphically demonstrated in Figure 1. Networks with a significantly increased standardized EOS ratio were Spain and Japan. Those with a significantly decreased ratio were Finland, Sweden, Brazil, Australia and New Zealand, and Canada.

### Mortality

**Figure 2:**
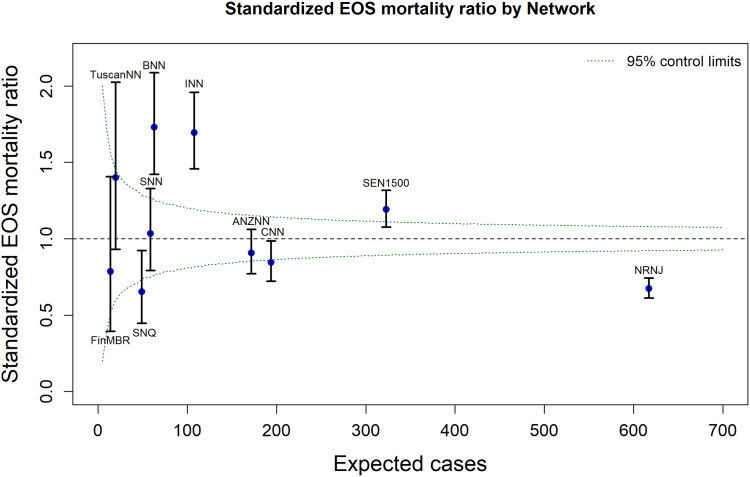
Comparison of standarized EOS mortality ratio with expected cases by network. Points represent standardized ratios for comparative analyses between networks using an indirect standardization approach. Error bars represent 95% confidence intervals of standardized ratios for each network and the dotted lines represent the 95% confidence intervals of standardized ratios based on expected cases. *ANZNN*, Australian and New Zealand Neonatal Network; *BNN*, Brazilian Network on Neonatal Research; *CNN*, Canadian Neonatal Network; FinMBR, Finnish Medical Birth Register; *INN*, Israel Neonatal Network; *NRNJ*, Neonatal Research Network of Japan; *SEN1500*, Spanish Neonatal Network; *SNN*, Swiss Neonatal Network; *SNQ*, Swedish Neonatal Quality Register; *TuscanNN*, Tuscany Neonatal Network.

Across the entire iNeo study population, the observed all-cause mortality rate among neonates diagnosed with EOS was 35.5%, with an AOR of 2.49 (95% CI, 2.27-2.74) compared to those without (Table 4). Mortality rates among neonates diagnosed with EOS varied between 22.5% in Sweden and 56.8% in Brazil. Within each network, all countries had a statistically significant increased adjusted odds of mortality among neonates with EOS compared to those without, except Finland (AOR, 1.89; 95% CI, 0.70-5.11) and Sweden (AOR, 1.65; 95% CI, 0.93-2.92), which were not statistically significantly different (Table 4). A comparison of standardized EOS mortality OR with expected cases by network is graphically demonstrated in Figure 2. Networks with a significantly increased standardized EOS mortality ratio were Brazil and Israel. The one with a significantly decreased standardized ratio was Japan.

**Table 4:** Outcomes of EOS by network

Country	EOS mortality Rate N/Total N (%)	EOS mortality Unadjusted oR (95% CI)	EOS mortality aOR (95% CI)	EOS mortality or morbidity rate N/Total N (%)	EOS mortality and/or morbidity Unadjusted oR (95% CI)	EOS mortality and/or morbidity aOR (95% CI)
ANZNN	156/512 (30.5)	2.9 (2.3-3.8)	2.3 (1.7-3.1)	391/512 (76.1)	1.8 (1.4-2.4)	1.4 (1.1-1.9)
BNN	109/192 (56.8)	1.7 (1.2-2.5)	1.9 (1.2-2.9)	165/192 (85.9)	2.2 (1.3-3.8)	2.2 (1.2-4.0)
CNN	164/560 (29.3)	2.4 (1.9-3.1)	2.0 (1.5-2.6)	421/560 (75.2)	1.8 (1.4-2.3)	1.5 (1.1-2.0)
FinMBR	11/44 (25.0)	1.5 (0.6-3.7)	1.9 (0.7-5.1)	22/44 (50.0)	1.3 (0.6-2.8)	1.3 (0.5-3.0)
INN	183/347 (52.7)	2.9 (2.2-3.9)	4.0 (2.8-5.6)	286/347 (81.8)	2.7 (1.8-3.8)	2.7 (1.8-4.0)
NRNJ	416/1235 (33.7)	5.9 (5.0-6.9)	4.6 (3.8-5.5)	986/1235 (79.8)	2.5 (2.1-3.0)	1.8 (1.5-2.2)
SEN1500	385/1092 (35.3)	1.7 (1.4-2.0)	1.7 (1.4-2.1)	897/1092 (82.1)	2.0 (1.6-2.4)	2.00 (1.6-2.5)
SNQ	32/142 (22.5)	1.8 (1.0-3.0)	1.7 (0.9-2.9)	99/177 (55.9)	1.8 (1.1-2.8)	1.8 (1.1-3.0)
SNN	61/177 (34.5)	3.3 (2.2-5.1)	2.8 (1.8-4.5)	93/142 (65.5)	2.1 (1.4-3.1)	1.7 (1.1-2.6)
Tuscan NN	28/53 (52.8)	3.6 (1.7-7.5)	3.3 (1.4-8.0)	45/53 (84.9)	4.4 (1.6-11.8)	3.6 (1.2-10.8)
Total Population	1545/4354 (35.5)	2.8 (2.5-3.0)	2.5 (2.3-2.7)	3405/4354 (78.2)	2.2 (2.0-2.4)	1.9 (1.7-2.1)

Neonatal registries: ANZNN (Australian and New Zealand Neonatal Network), BNN (Brazilian Network on Neonatal Research), CNN (Canadian Neonatal Network), FinMBR (Finnish Medical Birth Register), INN (Israel Neonatal Network), NRNJ (Neonatal Research Network of Japan), SEN1500 (Spanish Neonatal Network), SNN (Swiss Neonatal Network), SNQ (Swedish Neonatal Quality Register), TuscanNN (Tuscany Neonatal Network), iNeo (International Network for Evaluation of Outcomes in Neonates).

Odds ratios are adjusted for by gestational age, birth weight z-score, sex, multiple births, cesarean birth, and outborn.

### Mortality and Morbidity

**Figure 3:**
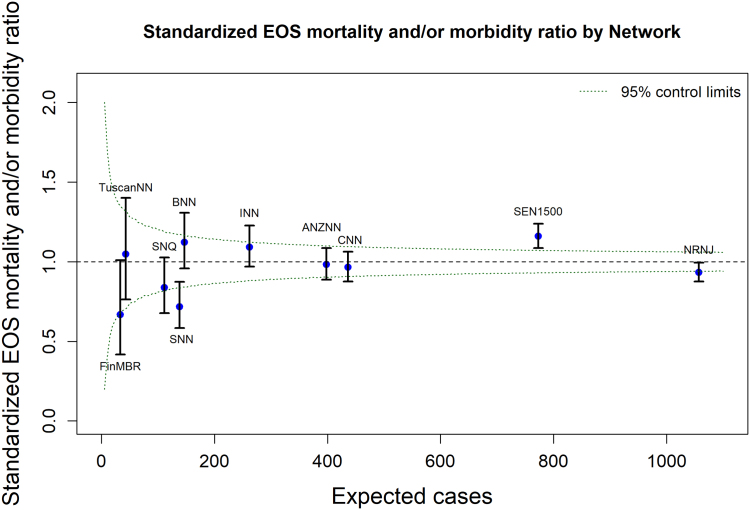
Comparison of standarized EOS mortality and/or morbidity ratio with expected cases by network. Points represent standardized ratios for comparative analyses between networks using an indirect standardization approach. Error bars represent 95% confidence intervals of standardized ratios for each network and the dotted lines represent the 95% confidence intervals of standardized ratios based on expected cases. *ANZNN*, Australian and New Zealand Neonatal Network; *BNN*, Brazilian Network on Neonatal Research; *CNN*, Canadian Neonatal Network; FinMBR, Finnish Medical Birth Register; *INN*, Israel Neonatal Network; *NRNJ*, Neonatal Research Network of Japan; *SEN1500*, Spanish Neonatal Network; *SNN*, Swiss Neonatal Network; *SNQ*, Swedish Neonatal Quality Register; *TuscanNN*, Tuscany Neonatal Network.

The rate of mortality and/or morbidity in the total population was 78.2%, with an AOR of 1.89 (95% CI, 1.70-2.10)) for neonates with EOS compared to those without EOS. When analyzed individually, the leading complication of EOS was severe neurological injury (AOR, 2.34; 95% CI, 2.11-2.59) with twice as many neonates diagnosed with EOS having severe neurological injury (40%) compared to those that were not diagnosed with EOS (20%) (Table 5). The rate of mortality and/or morbidity varied between 50.0% in Finland and 85.9% in Brazil. Statistically significant increased adjusted odds of mortality and/or morbidity among neonates with EOS were observed in all networks, except for Finland (AOR, 1.26; 95% CI: 0.54-2.97). A comparison of standardized EOS mortality and/or morbidity OR with expected cases by network is graphically demonstrated in Figure 3. Networks with a significantly increased standardized EOS ratio were Spain. There were no networks with a significantly decreased standardized ratio.

**Table 5:** Individual morbidity outcomes of EOS by total populations

Country	EOS N/Total N (%)	No EOS N/Total N (%)	Unadjusted aOR (95% CI)	aOR (95% CI)
Severe neurological injury	1263/3161 (40.0)	17523/70195 (20.0)	2.7 (2.4-2.9)	2.3 (2.1-2.6)
Treated retinopathy of prematurity	976/2741 (35.6)	20181/88716 (22.8)	1.9 (1.7-2.1)	1.7 (1.5-1.9)
Late onset sepsis	857/3633 (23.6)	27806/115100 (24.1)	1.0 (0.9-1.1)	0.8 (0.7-0.9)
Necrotising enterocolitis	398/4330 (9.2)	8616/124358 (6.9)	1.4 (1.2-1.6)	1.2 (1.1-1.4)
Bronchopulmonary dysplasia	179/332 (53.9)	4424/7834 (56.5)	0.9 (0.7-1.2)	0.7 (0.5-1.0)

Odds ratios are adjusted for by gestational age, birth weight z-score, sex, multiple births, cesarean birth, and outborn.

## Discussion

Across a multinational cohort of EP neonates born between 2007 and 2023, incidence rates of EOS remained high at 3.3% and consistent over time. There was variability in the incidence of EOS between countries. Spain had the highest rate and odds of EOS compared to the other networks and Finland had the lowest rate and odds of EOS comparatively. Additionally, approximately one out of 3 neonates diagnosed with EOS died and 3 out of 4 neonates either died or had major morbidity. Mortality and morbidity rates also varied between countries, with Brazil having the highest mortality rate and mortality and/or morbidity rate compared to other networks. Japan also stood out as having a reduced EOS mortality ratio compared to its expected cases. A recent survey on antimicrobial use for extreme preterm neonates diagnosed with EOS across different NICUs in iNeo demonstrated variances in antimicrobial regimes across different countries.^16^ Japanese NICUs particularly took a more conservative approach, with many units treating gram positive sepsis with 14 days of intravenous antibiotics and gram negative sepsis with 21 days.^16^ This may reflect their reduced mortality ratio, but a formal link could not be demonstrated in this study. Finland and Sweden data indicate that EOS was not associated with adjusted odds of mortality and mortality/morbidity. We speculate that this is possibly due to a lower incidence of EOS in these 2 networks which resulted in wide confidence intervals. However, it is also possible that outcomes truly do not differ in these networks between patients with and without EOS. Different countries have previously reported comparable EOS incidences and outcomes among preterm neonates, including networks within the iNeo database, such as ANZNN,^3^ INN,^17^ and CNN,^12^ as well as other epidemiologic studies in the United States,^2^ Norway,^18^ and Switzerland.^19^ However, it is difficult to compare these studies to the current study, given their differing exclusion criteria, including gestational age. It is also difficult to make any meaningful comparisons to current literature in Italy, Spain, Japan, and Brazil as these studies either did not differentiate between term and preterm neonates, focused on specific causative microorganisms or antibiotic resistance profiles or studied late-onset sepsis rather than EOS.^20^, ^21^, ^22^, ^23^ It is important to carefully interpret and compare these studies, as the baseline population drives the rate in each sample and can inflate or minimize results of outcomes. However, it can be concluded that incidence rates of EOS among EP neonates have not changed significantly over time.

Overall, severe neurological injury was observed to be the predominant complication of EOS, with twice as many neonates diagnosed with EOS having severe neurological injury compared to those that were not diagnosed with EOS. This is consistent with previously published analyses of smaller cohorts.^24^ Huang et al identified out of neonates born at less than 28 weeks’ gestation diagnosed with EOS, 41% were diagnosed with severe PVL or IVH compared to 24% of mild PVL or IVH.^25^ It has been theorized that IVH and PVL can develop secondary to the systemic inflammatory response resulting from bacteremia.^25^ The cascade of inflammatory cytokines can injure the blood brain barrier and cerebral vasculature that is already fragile among extremely preterm neonates, leading to vessel perforation. Additionally, the inflammatory response can hinder appropriate blood pressure regulation, in turn significantly altering cerebral perfusion, which is already known to be a contributing factor for IVH and PVL. Direct CNS infections, such as meningitis, can similarly predispose neonates to IVH and PVL. Beyond EOS, there are many contributing factors for neurological injury that are difficult to control for in such a large dataset. However, recent smaller studies in Germany^24^ and China^25^ have also demonstrated increased odds of developing IVH of varying degrees of severity due to the presence of EOS among preterm neonates using multivariate analyses.

Recent literature recognizes that EOS incidence is heavily influenced by intrapartum or maternal factors, such as spontaneous preterm labor, which may indicate reasons for variances of incidence between countries. Chorioamnionitis also has been found to not only be a risk factor for spontaneous preterm labor,^26^^,^^27^ but also increases the risk of EOS.^28^^,^^29^ Unfortunately, we do not have enough information on chorioamnionitis in our database to investigate further. However, it has been theorized that noninfective indications for preterm birth, such as pre-eclampsia, may place infants at lower risk of developing EOS.^2^ In medically-indicated preterm delivery, ∼90% are delivered via caesarean section without preterm labor,^30^ potentially highlighting a large cohort of neonates at lower risk of developing EOS. In our study, lower rates of EOS were detected among neonates born by caesarean section, with hypertension in pregnancy and as twins or high order. This has been consistent with previous literature in Australia, New Zealand, Israel, and the USA.^2^^,^^3^^,^^6^ However, in this study, higher rates of caesarean section, birth of twins or higher order or hypertension in pregnancy were not necessarily associated with lower rates of EOS within individual countries. Spain, with the highest rate and odds of EOS in the study, had a caesarean section rate of 61.4%, hypertension in pregnancy rate of 16.8% and birth of twins or higher order rate of 38.0%, which were similar to the overall population rates (65.0%, 11.9% and 26.5% respectively). Interestingly in comparison with Finland with the lowest rate and odds of EOS, Finland had lower rates of hypertension in pregnancy (7.3%), but similar rates of birth of twins or higher order (26.8%) and caesarean section rate (61.5%) to that in Spain. Identifying low- and high-risk preterm neonates for developing EOS continues to remain a challenge due to different risk factors from term neonates, and indiscernible clinical statuses between infected and non-infected preterm neonates.^31^^,^^32^ However, preterm neonates delivered via caesarean section without the presence of preterm labor and who are hemodynamically stable have been considered a cohort that can be targeted for the reduction of empirical antibiotic therapy.^31^ Further investigation into the preterm EOS risk due to these factors may further highlight the reasons for varying EOS incidences worldwide, but may also herald a new era of antibiotic stewardship.

Our study has several strengths. The large sample size acquired from a multinational database instills confidence that this study is a fairly accurate representation of the preterm population of each participating country. In addition, standardized definitions and data collection and continual benchmarking and quality improvement projects conducted by iNeo facilitators allow this study to generate in-depth analyses otherwise unable to be performed in these countries previously. Random variation was also accounted for with 17 years of data.

We acknowledge some limitations. Given not all registries recorded data on causative microorganisms for EOS, this paper could not comment on the microorganism profile of each country and the differences between each. Due to strict data collection guidelines of some participating networks, only neonates with a birth weight of <1500 grams were included in the study, therefore not encapsulating larger neonates possibly overinflating mortality and morbidity outcomes. Aside from differing maternal factors, causative microorganisms and antibiotic resistance profiles influencing variability between countries,^33^^,^^34^ it is important to note there are other organizational factors that can also influence these variabilities, including philosophy of care provision such as routine use of antimicrobial drugs for extremely preterm infants,^35^ health expenditure, resources and staffing, regionalization, and variable population coverage from each network,^8^^,^^36^ which limits how readers can interpret the results in this study.

In this multinational cohort study of 11 participating countries, the incidence of EOS among neonates born between 22 and 28 weeks’ gestation varied between 2 and 6%. Overall, across the iNeo network, EOS was associated with higher odds of mortality and composite of mortality or morbidity, severe neurological injury being the predominant complication. Mortality and morbidity outcomes also varied between countries. This study was able to analyze EOS outcomes that closely reflect the EP populations within each participating country, providing a new benchmark for quality improvement of prevention, diagnosis and management of EOS. The range of differing outcomes for EOS-diagnosed EP neonates in this cohort highlights the need for further research into novel and tailored diagnostic and management strategies.

## CRediT authorship contribution statement

**Husharn L. Duggan:** Writing – review & editing, Writing – original draft, Visualization, Validation, Software, Methodology, Investigation, Conceptualization. **Prakesh S. Shah:** Writing – review & editing, Visualization, Validation, Software, Project administration, Methodology, Investigation, Funding acquisition, Formal analysis, Data curation, Conceptualization. **Malcolm Battin:** Writing – review & editing, Methodology, Investigation, Data curation. **Gil Klinger:** Writing – review & editing, Methodology, Investigation, Data curation. **Mark Adams:** Writing – review & editing, Methodology, Investigation, Data curation. **Maximo Vento:** Writing – review & editing, Methodology, Investigation, Data curation. **Vieri Lastrucci:** Writing – review & editing, Methodology, Investigation, Data curation. **Ligia Maria Suppo Sousa Rugolo:** Writing – review & editing, Methodology, Investigation, Data curation. **Maria Regina:** Writing – review & editing, Methodology, Investigation, Data curation. **Stellan Håkansson:** Writing – review & editing, Methodology, Investigation. **Tetsuya Isayama:** Writing – review & editing, Methodology, Investigation, Data curation. **Mikael Norman:** Writing – review & editing, Methodology, Investigation, Data curation. **Satoshi Kusuda:** Writing – review & editing, Methodology, Investigation, Data curation. **Brian Reichman:** Writing – review & editing, Methodology, Investigation. **Liisa Lehtonen:** Writing – review & editing, Methodology, Investigation, Data curation. **Kjell Helenius:** Writing – review & editing, Methodology, Investigation, Data curation. **Kei Lui Kei:** Writing – review & editing, Supervision, Methodology, Investigation, Data curation, Conceptualization. **Kenneth Tan:** Writing – review & editing, Writing – original draft, Validation, Supervision, Project administration, Methodology, Investigation, Conceptualization.

## Declaration of Competing Interest

The International Network for Evaluating Outcomes of Neonates (iNeo) has been supported by the 10.13039/501100000024Canadian Institutes of Health Research [APR-126340 and PBN 150642 to P.S.S.]. The Australian and New Zealand Neonatal Network is predominantly funded by membership contributions from the participating centers. The Canadian Neonatal Network is supported by a team grant from the Canadian Institutes of Health Research [CTP 87518], and by the participating centers. The Finnish Medical Birth Register is governmentally funded and kept by the Canadian Institutes of Health Research (THL). The Israel Neonatal Network very low birth weight infant database is partially funded by the Israel Center for Disease Control and the Ministry of Health. The Neonatal Research Network of Japan is partly funded by a Health Labor Sciences Research Grant from the 10.13039/501100003478Ministry of Health, Labor and Welfare of Japan. The Spanish Neonatal Network is supported by funds from the Spanish Neonatal Society (SENeo). The Swedish Neonatal Quality Register is funded by the Swedish Government (10.13039/501100005348Ministry of Health and Social Affairs), the Swedish Association of Local Communities and Regions (SALAR) and the participating units. The Swiss Neonatal Network is partially funded by the participating units in the form of membership fees. This research was also supported by 10.13039/501100014365Instituto de Investigación Sanitaria Carlos III (Ministry of Science, Innovation and Universities, Kingdom of Spain) [FIS17/0131 to M.V.]; and RETICS funded by the PN 2018-2021 (Spain), ISCIII- Sub-Directorate General for Research Assessment and Promotion, and the European Regional Development Fund (ERDF) [RD16/0022]; and by grants from a regional agreement on clinical research (ALF) between Region Stockholm and Karolinska Institutet [RS2022-0674 to M.N.]. The funding body played no role in the design or conduct of the study; the collection, management, analysis, or interpretation of the data; the preparation, review, or approval of the manuscript; or the decision to submit the manuscript for publication.

The authors have no conflicts of interest relevant to this article to disclose.

## Supplementary Data

Online-only supplement

Data statement
