# Supplementary material for: Early-Onset Sepsis in Preterm Neonates of 22-28 Weeks’ Gestation: An International Cohort Study
Source: J Pediatr Clin Pract. 2026 May 14;21:200216. doi: 10.1016/j.jpedcp.2026.200216 (PMC13272568; doi:10.1016/j.jpedcp.2026.200216)
Supplement: Supplementary Table [file mmc1.docx]

**Supplementary Table:** **Characteristics of Participating Neonatal Networks in the iNeo**

|  | **ANZNN** | **BNN** | **CNN** | **FinMBR** | **INN** | **NRNJ** | **SEN1500** | **SNN** | **SNQ** | **TuscanNN** |
| --- | --- | --- | --- | --- | --- | --- | --- | --- | --- | --- |
| Units from which data are included^a^ | 56 | 20 | 28 | 30 | 27 | 73 | 61 | 12 | 28 | 24 |
| Tertiary neonatal units in the country or region^b^ | 29 | 911 NICU (Tertiary neonatal units (123), level I (178) and level II (610) | 28 | 5 | 23 | 93 | 50 | 9 | 7 | 7 |
| Variances in data collection and unit participation^a^ | All units from New Zealand participate in the ANZNN    All tertiary and few lower level units participate in the ANZNN from Australia | From 2014-2019 the same 20 public university units participated. In 2020, data refers 19/20 units.  Infants with BW ≥1500 grams birth weight excluded | All tertiary units participate in CNN | All units participate in FinMBR | All units participate in INN    Infants with >1500 grams birth weight are excluded | Not all units participate in NRNJ | Not all units participate in SEN1500 | All tertiary and large secondary (level IIB) participate in SNN | Only Skåne region included in this study    This region was only included post-2011 | All units participate in TuscanNN |
| Approximate no. of births per year^a^ | 300 000 (Australia)  60 000 (New Zealand) | 2 900 000 live births/year  40 000 VLBW live births/year | 350 000 | 60 000 | 160 000 | 1 080 000 | 480 000 | 80 000 | 90 000 | 30 000 |
| Proportion of infants in network compared with national birth statistics^b^ | 92.5% | 4-5% of VLBW infants/ year | 92.5% | 99.1% | 95.0% | 61.1% | 76.1% | 99.7% | 100% | 100% |
| Gross domestic product^a^ (per capita, $USD, 2014)^a^ | 61 925 (Australia),  44 342 (New Zealand) | 12 071 | 50 235 | 49 823 | 37 208 | 36 194 | 29 767 | 85 594 | 58 938 | 29 271 |
| Health expenditure^c^ (% of gross domestic product, 2013)^a^ | 9.44 (Australia)  9.74 (New Zealand) | 8.0 | 10.86 | 9.40 | 7.42 | 10.30 | 8.88 | 11.47 | 9.71 | 8.9 |

^a^Kelly LE, Shah PS, Håkansson S, Kusuda S, Adams M, Lee SK, et al. Perinatal health services organization for preterm births: A multinational comparison. J Perinatol. 2017;37(7):762-8.

^b^Helenius K, Sjörs G, Shah PS, Modi N, Reichman B, Morisaki N, et al. Survival in Very Preterm Infants: An International Comparison of 10 National Neonatal Networks. Pediatrics. 2017;140(6):e20171264.
