# Supplementary material for: Early-Onset Sepsis in Preterm Neonates of 22-28 Weeks’ Gestation: An International Cohort Study
Source: J Pediatr Clin Pract. 2026 May 14;21:200216. doi: 10.1016/j.jpedcp.2026.200216 (PMC13272568; doi:10.1016/j.jpedcp.2026.200216)
Supplement: Online-only supplement [file mmc2.docx]

**Online-only supplement**

[**List of investigators in iNeo networks** 2](#_Toc211533416)

**List of investigators in iNeo networks**

ANZNN (Australian and New Zealand Neonatal Network): Kei Lui* Chair of ANZNN. Flinders Medical Centre, SA: Scott Morris. Gold Coast University Hospital, QLD: Peter Schmidt, Manbir Chauhan*. Blacktown District Hospital, NSW: Anjali Dhawan*. John Hunter Children’s Hospital, NSW: Larissa Korostenski. King Edward Memorial and Perth Children’s Hospitals, WA: Mary Sharp, Tobias Strunk*. Liverpool Hospital, NSW: Jacqueline Stack. Mater Mothers' Hospital, QLD: Pita Birch, Tori Oliver*. Neonatal Retrieval Emergency Service Southern Queensland, QLD: Lucy Cooke. Mercy Hospital for Women, VIC: Dan Casalaz, Jim Holberton*. Monash Medical Centre, VIC: Alice Stewart, Rod Hunt*, Kenneth Tan*. Nepean Hospital, NSW: Lyn Downe. Paediatric Infant Perinatal Emergency Retrieval (VIC): Michael Stewart. NSW Newborn & Paediatric Emergency Transport Service: Andrew Berry. Royal Children's Hospital, VIC: Leah Hickey. Royal Darwin Hospital, NT: Mantho Kgosiemang, Laura Prado*. Royal Hobart Hospital, Tasmania: Tony De Paoli, Naomi Spotswood*. Royal Hospital for Women, NSW: Srinivas Bolisetty, Kei Lui*. Royal North Shore Hospital, NSW: Eveline Staub. Royal Prince Alfred Hospital, NSW: Mark Greenhalgh. Royal Brisbane and Women's Hospital, QLD: Pieter Koorts. Royal Women's Hospital, VIC: Risha Bhatia. SAAS MedSTAR Emergency Medical Retrieval Services: Bron Hennebry. Sydney Children's Hospital, NSW: Hari Ravindranathan. The Canberra Hospital, ACT: Hazel Carlisle. The Children's Hospital at Westmead, NSW: Nadia Badawi, Himanshu Popat. The Townsville Hospital, QLD: Gary Alcock. Neonatal Emergency Transport Service of Western Australia: Jonathan Davis. Westmead Hospital, NSW: Melissa Luig*. Women's & Children's Hospital, SA: Michael Stark. Australian College of Neonatal Nurses: Margaret Broom*. National Perinatal Epidemiology and Statistics Unit, University of New South Wales: Georgina Chambers*. Consumer representative: Natalie Merida*. New Zealand: Christchurch Women's Hospital: Bronwyn Dixon. University of Otago, Christchurch: Brian Darlow. Dunedin Hospital: Jason Wister. Middlemore Hospital: Kristin O’Connor. Auckland City Hospital: Mariam Buksh, Malcolm Battin*. Waikato Hospital: Jutta van den Boom. Wellington Women's Hospital: Angelica Allermo-Fletcher, Helen Miller. Whangarei Hospital: David Barker*. Neonatal Nurses College Aotearoa: Claire Jacobs*. Singapore: KK Women's and Children's Hospital, Singapore: Victor Samuel Rajadurai*. Hong Kong: Prince of Wales Hospital: Simon Lam. United Christian Hospital: Genevieve Fung. *denotes the ANZNN Executive Committee.

Brazilian Network on Neonatal Research (Rede Brasileira de Pesquisas Neonatais, RBPN): Ruth Guinsburg, RBPN Scientific Chair, Hospital São Paulo - EPM-UNIFESP, São Paulo, SP; Maria Fernanda de Almeida, RBPN Scientific Chair; José Maria de Andrade Lopes, Administrative Chair; Fábio Carmona, Database Coordination; Jamil Pedro de Siqueira Caldas, Data Collection Coordination; Silvia Cwajg, Instituto Fernandes Figueira – Fiocruz, Rio de Janeiro, RJ; Jorge Hecker Luz, Hospital São Lucas – PUCRS, Porto Alegre, RS; Humberto Holmer Fiori, Santa Casa de Porto Alegre, Porto Alegre, RS; Lígia Maria Suppo de Souza Rugolo, Hospital das Clínicas da Faculdade de Medicina de Botucatu – UNESP, Botucatu, SP; Renato Procianoy, Hospital de Clinicas de Porto Alegre – UFRGS, Porto Alegre, RS; Vera Lucia Jornada Krebs, Hospital das Clínicas – FMUSP, São Paulo; SP; Walusa Assad Gonçalves Ferri, Hospital das Clínicas de Ribeirão Preto – USP, Ribeirão Preto, SP; Sérgio Tadeu Martins Marba, Centro de Atenção a Saúde da Mulher – UNICAMP, Campinas, SP; José Luiz Bandeira Duarte, Hospital Universitário Pedro Ernesto – UERJ, Rio de Janeiro, RJ; Maria Albertina Santiago Rego, Hospital das Clínicas – UFMG, Belo Horizonte, MG; Regina Vieira Cavalcanti da Silva, Hospital das Clínicas – UFPR, Curitiba, PR; José Mariano Alves Junior, Maternidade Escola Hilda Brandão – FCMMG, Belo Horizonte, MG; Daniela Marques de Lima M Ferreira, Hospital das Clínicas – UFU, Uberlândia, MG; Lígia S Lopes Ferrari, Hospital Universitário – UEL, Londrina, PR; Jucille Meneses, Instituto de Medicina Integral Prof. Fernando Figueira – IMIP, Recife, PE; Marynea Silva do Vale, Hospital Universitário – UFMA, São Luís, MA; Edna Maria de Albuquerque Diniz, Hospital Universitário – USP, São Paulo, SP; Nathalia Moura de Mello e Silva, Hospital Estadual de Diadema - SPDM-UNIFESP, Diadema, SP; Milton Harumi Miyoshi, Hospital Geral de Pirajussara – SPDM-UNIFESP, Taboão da Serra, SP; Lais Furlan Embrizi, Hospital Estadual Sumaré – UNICAMP, Sumaré, SP.

CNN (Canadian Neonatal Network): Marc Beltempo, MD, (Director, Canadian Neonatal Network and Site Investigator), Montreal Children’s Hospital at McGill University Health Centre, Montréal, Québec; Prakesh S Shah, MD, MSc, Mount Sinai Hospital, Toronto, Ontario; Thevanisha Pillay, MD, Victoria General Hospital, Victoria, British Columbia; Jonathan Wong, MD, British Columbia Women’s Hospital, Vancouver, British Columbia; Miroslav Stavel, MD, Royal Columbian Hospital, New Westminster, British Columbia; Rebecca Sherlock, MD, Surrey Memorial Hospital, Surrey, British Columbia; Ayman Abou Mehrem, MD, Foothills Medical Centre, Calgary, Alberta; Jennifer Toye, MD, and Joseph Ting, MD, Royal Alexandra Hospital and University of Alberta Hospital, Edmonton, Alberta; Carlos Fajardo, MD, Alberta Children’s Hospital, Calgary, Alberta; Andrei Harabor, MD, Regina General Hospital, Regina, Saskatchewan; Lannae Strueby, MD, Jim Pattison Children’s Hospital , Saskatoon, Saskatchewan; Mary Seshia, MBChB, and Deepak Louis, MD, Winnipeg Health Sciences Centre, Winnipeg, Manitoba; Chelsea Ruth, MD, and Ann Yi, MD, St. Boniface General Hospital, Winnipeg, Manitoba; Amit Mukerji, MD, Hamilton Health Sciences Centre, Hamilton, Ontario; Orlando Da Silva, MD, MSc, London Health Sciences Centre, London, Ontario; Sajit Augustine, MD, Windsor Regional Hospital, Windsor, Ontario; Kyong-Soon Lee, MD, MSc, Hospital for Sick Children, Toronto, Ontario; Eugene Ng, MD, Sunnybrook Health Sciences Centre, Toronto, Ontario; Brigitte Lemyre, MD, The Ottawa Hospital, Ottawa, Ontario; Brigitte Lemyre, MD, Children’s Hospital of Eastern Ontario, Ottawa, Ontario; Faiza Khurshid, MD, Kingston General Hospital, Kingston, Ontario; Victoria Bizgu, MD, Jewish General Hospital, Montréal, Québec; Keith Barrington, MBChB, Anie Lapointe, MD, and Guillaume Ethier, NNP, Hôpital Sainte-Justine, Montréal, Québec; Christine Drolet, MD, dCentre Hospitalier Universitaire de Québec, Sainte Foy, Québec; Martine Claveau, MSc, LLM, NNP, Montreal Children’s Hospital at McGill University Health Centre, Montréal, Québec; Marie St-Hilaire, MD, Hôpital Maisonneuve-Rosemont, Montréal, Québec; Valerie Bertelle, MD, and Edith Masse, MD, Centre Hospitalier Universitaire de Sherbrooke, Sherbrooke, Québec; Caio Barbosa de Oliveira, MD, Moncton Hospital, Moncton, New Brunswick; Hala Makary, MD, Dr. Everett Chalmers Hospital, Fredericton, New Brunswick; Gabriela de Carvalho Nunes, MD, and Wissam Alburaki, MD, Saint John Regional Hospital, Saint John, New Brunswick; Jo-Anna Hudson, MD, Janeway Children’s Health and Rehabilitation Centre, St. John’s, Newfoundland; Jehier Afifi, MB BCh, MSc, IWK Health Centre, Halifax, Nova Scotia; Andrzej Kajetanowicz, MD, Cape Breton Regional Hospital, Sydney, Nova Scotia; Bruno Piedboeuf, MD (Chairman, Canadian Neonatal Network), Centre Hospitalier Universitaire de Québec, Sainte Foy, Québec.

FinMBR (Finnish Medical Birth Register): Marjo Metsäranta, MD, Helsinki University Hospital, Helsinki; Liisa Lehtonen, MD, Turku University Hospital, Turku; Päivi Korhonen, MD, Tampere University Hospital, Tampere; Ulla Sankilampi, MD, Kuopio University Hospital, Kuopio; Timo Saarela, MD, Oulu University Hospital, Oulu.

INN (Israel Neonatal Network): Iris Morag, MD, Assaf Harofeh Medical Center, Tzrifin; Omer Globus MD, Assuta Hospital, Ashdod; Shmuel Zangen, MD, Barzilai Medical Center, Ashkelon; Tatyana Smolkin, MD, Baruch Padeh Medical Center, Poriya; Yaron Nave, MD, Bikur Cholim Hospital, Jerusalem; Arieh Riskin, MD, Bnai Zion Medical Center, Haifa; Karen Lavie-Nevo, MD, Carmel Medical Center, Haifa; Zipora Strauss, Chaim Sheba Medical Center, Ramat Gan; Clari Felszer, MD, Emek Medical Center, Afula; Hussam Omari, MD, French Saint Vincent de Paul Hospital, Nazareth; Smadar Even Tov-Friedman, MD, Hadassah University Hospital-Ein Karem, Jerusalem; Smadar Even Tov-Friedman, MD, Hadassah University Hospital-Har Hazofim, Jerusalem; Amit Hochberg, MD, Hillel Yaffe Medical Center, Hadera; Nizar Saad, MD, Holy Family (Italian) Hospital, Nazareth; Orna Flidel-Rimon, MD, Kaplan Medical Center, Rehovot; Aryeh Simmonds, MD, Laniado Hospital, Netanya; Bernard Barzilay, MD, Mayanei Hayeshua Medical Center, Bnei Brak; Sofia Bauer, MD, Meir Medical Center, Kfar Saba; Amir Kugelman, MD, Rambam Medical Center; Eric Shinwell, MD, Rivka Ziv Medical Center, Safed; Gil Klinger, MD, Schneider Children’s Medical Center of Israel, Rabin Medical Center (Beilinson Campus), Petah Tikva; Yousif Nijim, MD, Scottish (EMMS) Hospital, Nazareth; Yaron Nave, MD, Shaare-Zedek Medical Center, Jerusalem; Eilon Shani, MD, Soroka Medical Center, Beersheba; Dror Mandel, MD, Sourasky Medical Center, Tel Aviv; Vered Fleisher-Sheffer, MD,Western Galilee Medical Center, Nahariya; Anat Oron, MD, Wolfson Medical Center, Holon; Lev Bakhrakh, MD, Yoseftal Hospital, Eilat.

NRNJ (Neonatal Research Network of Japan): Yukiteru Tachibana, MD, Abashiri Kosei Hospital, Abashiri, Hokkaido; Ayumu Noro, MD, JCHO Hokkaido Hospital, Sapporo, Hokkaido; Toshihiko Mori, MD, NTT East Sapporo Hospital, Sapporo, Hokkaido; Ken Nagaya, MD, Asahikawa Medical University, Asahikawa, Hokkaido; Masaru Shirai, MD, Asahikawa Kosei Hospital, Asahikawa, Hokkaido; Toru Ishioka, MD, Engaru Kosei Hospital, Engaru, Hokkaido; Toshiya Saito, MD, Iwamizawa City Hospital, Iwamizawa, Hokkaido; Yosuke Kaneshi, MD, Kushiro Red Cross Hospital, Kushiro, Hokkaido; Masaki Kobayashi, MD, Sapporo Prefecture Medical University, Sapporo, Hokkaido; Nobuko Shiono, MD, Sapporo City Hospital, Sapporo, Hokkaido; Nobuhiro Takahashi, MD, Tenshi Hospital, Sapporo, Hokkaido; Yusuke Ohkado, MD, Tomakomai Chity Hospital, Tomakomai, Hokkaido; Tatsuro Satomi, MD, Nikko Kinen Hospital, Sapporo, Hokkaido; Mika Nakajima, MD, Hakodate Central Hospital, Hakodate, Hokkaido; Eiki Nakamura, MD, Nayoro City Hospital, Nayoro, Hokkaido; Tomofumi Ikeda, MD, Aomori Prefecture Central Hospital, Aomori, Aomori; Genichiro Sotodate, MD, Iwate Medical University, Morioka, Iwate; Mari Ishii, MD, Iwate Prefecture Kuji Hospital, Kuji, Iwate; Toru Huchimukai, MD, Iwate Prefecture Ohfunato Hospital, Ohfunato, Iwate; Takahide Hosokawa, MD, Iwate Prefecture Ninohe Hospital, Ninohe, Iwate; Rikio Suzuki, MD, Sendai City Hospital, Sendai, Miyagi; Masatoshi Sanjo, MD, Sendai Red Cross Hospital, Sendai, Miyagi; Michiya Kudo, MD, Osaki City Hospital, Osaki, Iwate; Takushi Hanita, MD, Tohoku University, Sendai, Miyagi; Hirokazu Arai, MD, Akita Red Cross Hospital, Akita, Akita; Masato Ito, MD, Akita University, Akita, Akita; Satoshi Niwa, MD, Odate City Hospital, Odate, Akita; Masanari Kawamura, MD, Hiraka General Hospital, Hiraka, Akita; Satoshi Watanabe, MD, Yamagata Prefecture Central Hospital, Yamagata, Yamagata; Yousuke Sudo, MD, Yamagata University, Yamagata, Yamagata; Hiroshi Yoshida, MD, Tsuruoka City Shonai Hospital, Tsuruoka, Yamagata; Tsutomu Ishii, MD, National Fukushima Hospital, Sukagawa, Fukushima; Takashi Imamura, MD, Takeda General Hospital, Aizuwakamatsu, Fukusima; Maki Sato, MD, Fukusima Prefecture Medical University, Fukushima, Fukushima; Yoshiya Yukitake, MD, Ibaraki Children's Hospital, Mito, Ibaraki; Yayoi Miyazono, MD, Tsukuba University, Tsukuba, Ibaraki; Goro Asada, MD, Tsuchiura Kyodo Hospital, Tsuchiura, Ibaraki; Yumi Kono, MD, Jichi Medical University, Oyama, Tochigi; Yasuaki Kobayashi, MD, Ashikaga Red Cross Hospital, Ashikaga, Tochigi; Hiroshi Suzuki, MD, Dokkyo Medical University, Shimotsugagun, Tochigi; Yasushi Oki, MD, Kiryu Kosei General Hospital, Kiryu, Gunma; Kenji Ichinomiya, MD, Gunma Prefecture Children's Hospital, Maebashi, Gunma; Toru Fujiu, MD, Gunma University, Maebashi, Gunma; Hideaki Fukushima, MD, Ohta General Hospital, Ota, Gunma; Hideshi Fujinaga, MD, National Nishisaitama Central Hospital, Tokorozawa, Saitama; Tetsuya Kunikata, MD, Saitama Medical University, Iruma, Saitama; Fumihiko Namba, MD, Saitama Medical University Medical Center, Kawagoe, Satitama; Masaki Shimizu, MD, Saitama Prefecture Children's Hospital, Omiya, Saitama; Shigeharu Hosono, MD, Jichi Medical University Saitama Medical Center, Omiya, Saitama; Chika Morioka, MD, Kawaguchi City Medical Center, Kawaguchi, Saitama; Motoichiro Sakurai, MD, Kameda General Hospital, Kameda, Chiba; Hiroshi Matsumoto, MD, Asahi Central Hospital, Asahi, Chiba; Naoto Nishizaki, MD, Juntendo University Urayasu Hospital, Urayasu, Chiba; Satoshi Toishi, MD, Narita Red Cross Hospital, Narita, Chiba; Harumi Otsuka, MD, Chiba City Kaihin Hospital, Chiba, Chiba; Masahiko Sato, MD, Tokyo Women's Medical University Yachiyo Medical Center, Yachiyo, Chiba; Kenichiro Hirakawa, MD, Aiiku Hospital, Minato, Tokyo; Kenichiro Hosoi, MD, Kyorin Univesity, Mitaka, Tokyo; Keiji Goishi, MD, National International Medical Center, Shinjuku, Tokyo; Yuji Ito, MD, National Center for Child Health and Development, Setagaya, Tokyo; Kyone Ko, MD, Sanikukai Hospital, Sumida, Tokyo; Hiromichi Shoji, MD, Juntendo University, Bunkyo, Tokyo; Atsuo Miyazawa, MD, Showa University, Shinagawa, Tokyo; Yuko Nagaoki, MD, Saint Luku Hospital, Chuo, Tokyo; Naoki Ito, MD, Teikyo University, Itabashi, Tokyo; Ken Masunaga, MD, Tokyo Metropolitan Otsuka Hospital, Toshima, Tokyo; Reiko Kushima, MD, Tokyo Metropolitan Bokuto Hospital, Sumida, Tokyo; Sakae Kumasaka, MD, Tokyo Katsushika Red Cross Perinatal Center, Katsushika, Tokyo; Manabu Sugie, MD, Tokyo Medical and Dental University, Bunkyo, Tokyo; Daisuke Haruhara, MD, Tokyo Medical University, Shinjuku, Tokyo; Masahiro Kobayashi, MD, Tokyo Jikei Medical University, Minatoku, Tokyo; Satsuki Kakiuchi, MD, Tokyo Women's Medical University, Shinjuku, Tokyo; Riki Nishimura, MD, Tokyo University, Bunkyo, Tokyo; Kaoru Okazaki, MD, Tokyo Metropolitan Children's Medical Center, Fuchu, Tokyo; Hitoshi Yoda, MD, Toho University, Ota, Tokyo; Atsushi Nakao, MD, Japan Red Cross Hospital, Shibuya, Tokyo; Ichiro Morioka, MD, Nihon University, Itabashi, Tokyo; Daisuke Ogata, MD, Yokohama City Hospital, Yokohama, Kanagawa; Fumihiko Ishida, MD, Yokohama City University Medical Center, Yokohama, Kanagawa; Daisuke Nishi, MD, Yokohama Rosai Hospital, Yokohama, Kanagawa; Miho Sato, MD, Yokosuka Kyosai Hospital, Yokosuka, Kanagawa; Ayako Fukuyama, MD, Yokohama Medical Center, Yokohama, Kanagawa; Kuriko Nakamura, MD, Saiseikai Eastern Yokohama Hospital, Yokohama, Kanagawa; Kanji Ogo, MD, Odawara City Hospital, Odawara, Kanagawa; Masahiko Murase, MD, Showa University Northern Yokohama Hospital, Yokohama, Kanagawa; Katsuaki Toyoshima, MD, Kanagawa Children's Medical Center, Yokohama, Kanagawa; Isamu Hokuto, MD, St. Marianna Medical University, Kawasaki, Kanagawa; Maha Suzuki, MD, St. Mariana Medical University Yokohama City Seibu Hospital, Yokohama, Kanagawa; Atsushi Uchiyama, MD, Tokai University, Isehara, Kanagawa; Yoshio Shima, MD, Nippon Medical School Musashi Kosugi Hospital, Kawasaki, Kanagawa; Hidehiko Nakanishi, MD, Kitasato University Hospital, Sagamihara, Kanagawa; Atsushi Nemoto, MD, Yamanashi Prefecture Central Hospital, Kofu, Yamanashi; Akira Shimazaki, MD, National Shinshu Ueda Medical Center, Ueda, Nagano; Tatsuya Yoda, MD, Saku General Hospital, Saku, Nagano; Yukihide Miyosawa, MD, Shinshu University, Matsumoto, Nagano; Takehiko Hiroma, MD, Nagano Children's Hospital, Azumino, Nagano; Yosuke Shima, MD, Iida City Hospital, Iida, Nagano; Gen Kuratsuji, MD, Niigata Central Hospital, Niigata, Niigata; Yoshihisa Nagayama, MD, Niigata City Hospital, Niigata, Niigata; Tohei Usuda, MD, Niigata University, Niigata, Niigata; Rei Kobayashi, MD, Nagaoka Red Cross Hospital, Nagaoka, Niigata; Hiroaki Imamura, MD, Koseiren Takaoka Hospital, Takaoka, Toyama; Takeshi Hutani, MD, Toyama Prefectural Central Hospital, Toyama, Toyama; Taketoshi Yoshida, MD, Toyama University, Toyama, Toyama; Azusa Kobayashi, MD, Kanazawa Medical University, Kanazawa, Kanazawa; Kazuhide Ohta, MD, Kanazawa Medical Center, Kanazawa, Kanazawa; Shuya Nagaoki, MD, Kanazawa University, Kanazawa, Ishikawa; Yasuhisa Ueno, MD, Ishikawa Prefectural Central Hospital, Kanazawa, Ishikawa; Toru Ando, MD, Tsuruga City Hospital, Tsuruga, Fukui; Ritsuyo Taguchi, MD, Fukui Prefectural Hospital, Fukui, Fukui; Takashi Okuno, MD, Fukui University, Fukui, Fukui; Hiroshi Yamamoto, MD, Gifu Prefectural Medical Center, Gifu, Gifu; Takeshi Arakawa, MD, Gifu Prefecture Tajimi Hospital, Tajimi, Gifu; Shinji Usui, MD, Takayama Red Cross Hospital, Takayama, Gifu; Yasushi Uchida, MD, National Nagara Medical Center, Nagara, Gifu; Takashi Tachibana, MD, Oogaki City Hospital, Oogaki, Gifu; Tokuso Murabayashi, MD, Numazu City Hospital, Numazu, Shizuoka; Tadayuki Kumagai, MD, Yaizu City Hospital, Yaizu, Shizuoka; Shigeru Oki, MD, Seirei Hamamatsu Hospital, Hamamatsu, Shizuoka; Reiji Nakano, MD, Shizuoka Children's Hospital, Shizuoka, Shizuoka; Taizo Ueno, MD, Shizuoka Saiseikai Hospital, Shizuoka, Shizuoka; Mitsuhiro Ito, MD, Fujieda City Hospital, Fujieda, Shizuoka; Masami Shirai, MD, Iwata City Hospital, Shizuoka, Shizuoka; Akira Oishi, MD, Hamamatsu Medical University, Hamamatsu, Shizuoka; Hikaru Yamamoto, MD, Toyota Memorial Hospital, Toyota, Aichi; Hiroshi Takeshita, MD, Aichi Medical University, Nagoya, Aichi; Yuichi Kato, MD, Anjokosei Hospital, Anjo, Aichi; Masashi Hayashi, MD, Okazaki City Hospital, Okazaki, Aichi; Kuniko Ieda, MD, Koritsu Tosei Hospital, Toyota, Aichi; Koji Takemoto, MD, Konankosei Hospital, Kona, Aichi; Takako Hirooka, MD, Komaki City Hospital, Komaki, Aichi; Masashi Miyata, MD, Fujita Medical University, Nagoya, Aichi; Makoto Ohshiro, MD, Japanese Red Cross Aichi Medical Center Nagoya Daiichi Hospital, Nagoya, Aichi; Masanori Kowaki, MD, Japanese Red Cross Aichi Medical Center Nagoya Daini Hospital, Nagoya, Aichi; Osamu Shinohara, MD, Handa City Hospital, Handa, Aichi; Yasunori Koyama, MD, Toyohashi City Hospital, Toyohashi, Aichi; Osuke Iwata, MD, Nogoya City University, Nagoya, Aichi; Takahiro Muramatsu, MD, Nagoya City Seibu Medical Cneter, Nagoya, Aichi; Akinobu Taniguchi, MD, Nagoya University, Nagoya, Aichi; Naoki Kamata, MD, Ise Red Cross Hospital, Ise, Mie; Hiroshi Uchizono, MD, National Mie Central Medical Center, Tsu, Mie; Kanemasa Maki, MD, Yokkaichi City Hospital, Yokkaichi, Mie; Takahide Yanagi, MD, Shiga Medical University, Otsu, Shiga; Kenji Nakamura, MD, Otsu Red Cross Hospital, Otsu, Shiga; Masahito Yamamoto, MD, Nagahama Red Cross Hospital, Nagahama, Shiga; Jitsuko Ohira, MD, Uji Tokushukai Hospital, Uji, Kyoto; Machiko Sawada, MD, Kyoto Katsura Hospital, Kyoto, Kyoto; Kozue Shiomi, MD, Kyoto City Hospital, Kyoto, Kyoto; Ryosuke Araki, MD, Kyoto University, Kyoto, Kyoto; Daisuke Kinoshita, MD, Kyoto Red Cross Daiichi Hospital, Kyoto, Kyoto; Ryuji Hasegawa, MD, Kyoto Prefecture Medical University, Kyoto, Kyoto; Akira Nishimura, MD, Kyoto Prefectural Medical University Northern Hospital, Yosagun, Kyoto; Hiroshi Komatsu, MD, National Maizuru Medical Center, Maizuru, Kyoto; Koji Nozaki, MD, Mitubishi Kyoto Hospital, Kyoto, Kyoto; Shinsuke Adachi, MD, Fukuchiyama City Hospital, Fukuchiyama, Kyoto; Toru Yamakawa, MD, Japan Baptist Hospital, Kyoto, Kyoto; Masahiko Kai, MD, Bell Land General Hospital, Sakai, Osaka; Hiroshi Sumida, MD, Rinku General Hospital, Izumisano, Osaka; Hirotaka Minami, MD, Takatsuski General Hospital, Takatsuki, Osaka; Kenji Mine, MD, Kansai Medical University, Hirakata, Osaka; Reiko Negi, MD, National Cerebral and Cardiovascular Center, Suita, Osaka; Satoru Ogawa, MD, Saiseikai Suita Hospital, Suita, Osaka; Ryoko Yoshinare, MD, Hannan Central Hospital, Hannan, Osaka; Yasuyuki Tokunaga, MD, Toyonaka City Hospital, Toyonaka, Osaka; Kiyoaki Sumi, MD, Aizenbashi Hospital, Osaka, Osaka; Akihiro Takatera, MD, Chifune Hospital, Osaka, Osaka; Atsushi Ogihara, MD, Osaka Medical University, Takatsuki, Osaka; Satoshi Onishi, MD, Osaka Metropolitan University, Osaka, Osaka; Taho Kim, MD, Osaka City Sumiyoshi Hospital, Osaka, Osaka; Hiroyuki Ichiba, MD, Osaka City General Hospital, Osaka, Osaka; Misao Yoshii, MD, Osaka Red Cross Hospital, Osaka, Osaka; Hitomi Okabe, MD, Osaka University, Suita, Osaka; Yoshio Kusumoto, MD, Osaka General Medical Center, Osaka, Osaka; Shinya Hirano, MD, Osaka Women's and Children's Hospital, Izumi, Osaka; Hiroshi Mizumoto, MD, Kitano Hospital, Osaka, Osaka; Yae Michinomae, MD, Yao City Hospital, Yao, Osaka; Makoto Nabetani, MD, Yodogawa Christian Hospital, Osaka, Osaka; Takeshi Morisawa, MD, Kakogawa City Hospital, Kakogawa, Hyogo; Masaaki Ueda, MD, Toyooka General Hospital, Toyooka, Hyogo; Takahiro Okutani, MD, Saiseikai Hyogo Hospital, Kobe, Hyogo; Masaru Yamakawa, MD, Kobe City Medical Center Central Hospital, Kobe, Hyogo; Kazumichi Fujioka, MD, Kobe University, Kobe, Hyogo; Tomoaki Ioroi, MD, Himeji Red Cross Hospital, Himeji, Hyogo; Takeshi Utsunomiya, MD, Hyogo Medical University Hospital, Nishinomiya, Hyogo; Seiji Yoshimoto, MD, Kobe Children's Hospital, Kobe, Hyogo; Tamaki Ohashi, MD, Hyogo Prefectural Awaji Medical Center, Sumoto, Hyogo; Toshiya Nishikubo, MD, Nara Prefecture Medical University, Kashiwara, Nara; Ken Kumagaya, MD, Wakayama Prefecture Medical University, Wakayama, Wakayama; Akiko Tamura, MD, Tottori Prefectural Central Hospital, Tottori, Tottori; Masumi Miura, MD, Tottori University, Yonago, Tottori; Yuki Hasegawa, MD, Matsue Red Cross Hospital, Matsue, Matsue; Rie Kanai, MD, Shimane Prefectural Central Hospital, Izumo, Shimane; Kei Takemoto, MD, Okayama Red Cross Hospital, Okayama, Okayama; Koichi Tsukamoto, MD, Okayama University, Okayama, Okayama; Misao Kageyama, MD, National Okayama Medical Center, Okayama, Okayama; Takashi Nakano, MD, Kawasaki Medical University, Kurashiki, Okayama; Hironobu Tokumasu, MD, Kurashiki Central Hospital, Kurashiki, Okayama; Moriharu Sugimoto, MD, Tsuyama Central Hospital, Tsuyama, Okayama; Rie Fukuhara, MD, Hiroshima Prefectural Hospital, Hiroshima, Hiroshima; Yutaka Nishimura, MD, Hiroshima City Central Hospital, Hiroshima, Hiroshima; Seiichi Hayakawa, MD, Hiroshima University, Hiroshima, Hiroshima; Yasuhiko Sera, MD, National Kure Medical Center, Kure, Hiroshima; Masahiro Tahara, MD, Tsuchiya General Hospital, Hiroshima, Hiroshima; Shinosuke Fukunaga, MD, Saiseikai Shimonoseki General Hospital, Shimonoseki, Yamaguchi; Keiko Hasegawa, MD, Yamaguchi Prefecture Medical Center, Hofu, Yamaguchi; Kazumasa Takahashi, MD, Yamaguchi University, Ube, Yamaguchi; Hiroshi Tateishi, MD, Tokuyama Central Hospital, Tokuyama, Yamaguchi; Tomomasa Terada, MD, Tokushima Prefecture Central Hospital, Tokushima, Tokushima; Takashi Yamagami, MD, Tokushima City Hospital, Tokushima, Tokushima; Takahiko Saijo, MD, Tokushima University, Tokushima, Tokushima; Kosuke Koyano, MD, Kagawa University, Kida, Kagawa; Toru Kuboi, MD, Shikoku Medical Center for Children and Adults, Zentsuji, Kagawa; Osamu Matsuda, MD, Ehime Prefectural Imabari Hospital, Imabari, Ehime; Shinosuke Akiyoshi, MD, Ehime Prefectural Central Hospital, Matsuyama, Ehime; Takahiro Motoki, MD, Uwajima City Hospital, Uwajima, Ehime; Yoichi Kondo, MD, Matsuyama Red Cross Hospital, Matsuyama, Ehime; Yusei Nakata, MD, Kochi Health Science Center, Kochi, Kochi; Hideaki Harada, MD, Kurume University, Kurume, Fukuoka; Masayuki Ochiai, MD, Kyushu University, Fukuoka, Fukuoka; Toshinori Nakashima, MD, National Kokura Medical Center, Kitakyushu, Fukuoka; Toshiharu Hikino, MD, National Kyushu Medical Center, Fukuoka, Fukuoka; Shutaro Suga, MD, University of Occupational and Environmental Health Japan, Kitakyushu, Fukuoka; Mitsuaki Unno, MD, Saint Maria Hospital, Kurume, Fukuoka; Hiroshi Kanda, MD, Iizuka Hospital, Iizuka, Fukuoka; Yasushi Takahata, MD, Fukuoka City Children's Hospital, Fukuoka, Fukuoka; Hiroyasu Kawano, MD, Fukuoka University, Fukuoka, Fukuoka; Takayuki Kokubo, MD, Kitakyushu City Hospital, Kitakyushu, Fukuoka; Toshimitsu Takayanagi, MD, National Saga Hospital, Saga, Saga; Mikio Aoki, MD, National Nagasaki Medical Center, Nagasaki, Nagasaki; Muneichiro Sumi, MD, Sasebo City Hospital, Sasebo, Nagasaki; Fumiko Kinoshita, MD, Nagasaki City Hospital, Nagasaki, Nagasaki; Tsutomu Ogata, MD, Nagasaki University, Nagasaki, Nagasaki; Kei Inomata, MD, Kumamoto City Hospital, Kumamoto, Kumamoto; Masanori Iwai, MD, Kumamoto University, Kumamoto, Kumamoto; Naoki Fukushima, MD, Almeida Memorial Hospital, Oita, Oita; Koichi Iida, MD, Oita Prefectural Hospital, Oita, Oita; Mitsushi Goshi, MD, Nakatsu City Hospital, Nakatsu, Oita; Yuki Kodama, MD, Miyazaki University, Miyazaki, Miyazaki; Shuichi Yanagibe, MD, National Miyakonojo Hospital, Miyakonojo, Miyazaki; Yuko Maruyama, MD, Imakyure General Hospital, Kagoshima, Kagoshima; Takuya Tokuhisa, MD, Kagoshima City Hospital, Kagoshima, Kagoshima; Yoriko Kisato, MD, Okinawa Prefectural Central Hospital, Uruma, Okinawa; Tatsuo Oshiro, MD, Okinawa Prefectural Nanbu Medical Center/Nanbu Child Medical Center, Shimajiri, Okinawa; Kazuhiko Nakasone, MD, Okinawa Red Cross Hospital, Naha, Okinawa; Asao Yara, MD, Naha City Hospital, Naha, Okinawa.

SEN1500 (Spanish Neonatal Network): Alejandro Avila-Alvarez, MD, and José Luis Fernandez-Trisac, MD, Complexo Hospitalario Universitario De A Coruña, A Coruña; Mª Luz Couce Pico, MD, and María José Fernández Seara, MD, Hospital Clínico Universitario de Santiago, Santiago de Compostela; Andrés Martínez Gutiérrez, MD, Complejo Hospitalario Albacete, Albacete; Carolina Vizcaíno , MD, Hospital General Universitario de Elche, Alicante; Miriam Salvador Iglesias, MD, and Honorio Sánchez Zaplana, MD, Hospital General Universitario de Alicante, Alicante; Belén Fernández Colomer, MD, and José Enrique García López, MD, Hospital Universitario Central de Asturias, Oviedo, Asturias; Rafael García Mozo, MD, and M. Teresa González Martínez, MD, Hospital Universitario de Cabueñes, Gijón, Asturias; Mª Dolores Muro Sebastián, MD, and Marta Balart Carbonell, MD, Clínica Corachán, Barcelona; Joan Badia Barnusell, MD, and Mònica Domingo Puiggròs, MD, Corporacio Parc Taulí, Sabadell, Barcelona; Josep Figueras Aloy, MD, and Francesc Botet Mussons, MD, Hospital Clínic de Barcelona, Barcelona; Israel Anquela Sanz, MD, Hospitalario De Granollers, Granollers; Gemma Ginovart Galiana, MD, H. De La Santa Creu I Sant Pau, Barcelona; W. Coroleu, MD, Hospital Universitari Germans Trias I Pujol, Badalona; Martin Iriondo, MD, Hospital Sant Joan de Déu Barcelona, Esplugues de Llobregat, Barcelona; Laura Castells Vilella, MD, Hospital General de Cataluña, Barcelona; Roser Porta, MD, Institute Dexeus, Barcelona; Xavier Demestre, MD, and Silvia Martínez Nadal, MD, Scias-Hospital Barcelona, Barcelona; Cristina de Frutos Martínez, MD, Hospital Universitario de Burgos, Burgos; María Jesús López Cuesta, MD, H. San Pedro de Alcántara, Cáceres; Dolores Esquivel Mora, MD, and Joaquín Ortiz Tardío, MD, Hospital Jerez, Cádiz; Isabel Benavente, MD, and Almudena Alonso, MD, Hospital Universitario Puerta Del Mar, Cádiz; Ramón Aguilera Olmos, MD, Hospital General de Castellón, Castellón; Miguel A. García Cabezas, MD, and Mª Dolores Martínez Jiménez, MD, Hospital General Universitario de Ciudad Real, Ciudad Real; Mª Pilar Jaraba Caballero, MD, and Mª Dolores Ordoñez Díaz, MD, Hospital Universitario Reina Sofía, Córdoba; Alberto Trujillo Fagundo, MD, and Lluis Mayol Canals, MD, Hospital Universitari de Girona Dr. Josep Trueta, Girona; Fermín García-Muñoz Rodrigo, MD, and Lourdes Urquía Martí, MD, H.M.I. Las Palmas, Las Palmas, Gran Canaria; María Fernanda Moreno Galdo , MD, and José Antonio Hurtado Suazo, MD, Hospital Universitario Virgen De Las Nieves, Granada; Eduardo Narbona López, and José Uberos Fernández, MD, Hospital Universitario San Cecilio, Granada; Miguel A Cortajarena Altuna, MD, and Oihana Muga Zuriarrain Hospital, MD, Donostia, Gipuzkoa; David Mora Navarro, MD, Hospital Juan Ramón Jiménez, Huelva; María Teresa Domínguez, MD, Hospital Costa De La Luz, Huelva; Mª Yolanda Ruiz del Prado, MD, and Inés Esteban Díez, MD, Hospital San Pedro, Logroño, La Rioja; María Teresa Palau Benavides, MD, and Santiago Lapeña, MD, Hospital de León, León, León; Teresa Prada, MD, Hospital del Bierzo, Ponferrada, León; Eduard Soler Mir, MD, Hospital Arnau De Vilanova, Lleida; Araceli Corredera Sánchez, MD, Enrique Criado Vega, MD, Náyade del Prado, MD, and Cristina Fernández, MD, Hospital Clínico San Carlos, Madrid; Lucía Cabanillas Vilaplana, MD, and Irene Cuadrado Pérez, MD, Hospital Universitario De Getafe, Madrid; Luisa López Gómez, MD, Hospital De La Zarzuela, Madrid; Laura Domingo Comeche, MD, Hospital Universitario de Fuenlabrada, Fuenlabrada, Madrid; Isabel Llana Martín, MD, Hospital Madrid-Torrelodones, Madrid, Madrid; Carmen González Armengod, MD, and Carmen Muñoz Labián, MD, Hospital Universitario Puerta De Hierro, Majadahonda, Madrid; Mª José Santos Muñoz, MD, Hospital Severo Ochoa, Leganés, Madrid; Dorotea Blanco Bravo, MD, and Vicente Pérez, MD, Hospital Gregorio Marañón, Madrid; Mª Dolores Elorza Fernández, MD, Celia Díaz González, MD, and Susana Ares Segura, MD, H.U. La Paz, Madrid; Manuela López Azorín, MD, Hospital Universitario Quirónsalud Madrid, Madrid; Ana Belén Jimenez MD, Hospital Universitario Fundación Jiménez Díaz, Madrid; Tomás Sánchez-Tamayo, MD, and Elías Tapia Moreno, MD, Hospital Carlos Haya, Málaga; María González, MD, and José Enrique Sánchez Martínez, MD, Hospital Parque San Antonio De Málaga, Málaga; José María Lloreda García, MD, Hospital Universitario Santa Lucia De Cartagena, Murcia; Concepción Goñi Orayen, MD, Hospital Virgen Del Camino De Pamplona, Pamplona, Navarra; Javier Vilas González, MD, Complexo Hospitalario Pontevedra, Pontevedra; María Suárez Albo, MD, and Eva González Colmenero, MD, Hospital Xeral De Vigo, Pontevedra; Elena Pilar Gutiérrez González, MD, and Beatriz Vacas del Arco, MD, Hospital Universitario de Salamanca, Salamanca; Josefina Márquez Fernández, MD, and Laura Acosta Gordillo, MD, Hospital Valme, Sevilla; Mercedes Granero Asensio, MD, Hospital Virgen De La Macarena, Sevilla; Carmen Macías Díaz, MD, Hospital Universitario Virgen Del Rocío, Sevilla; Mar Albújar, MD, Hospital Universitari de Tarragona Joan XXIII, Tarragona; Pedro Fuster Jorge. MD, Hospital Universitario De Canarias, San Cristóbal de La Laguna, Santa Cruz de Tenerife; Sabina Romero, MD, and Mónica Rivero Falero, MD, Hospital Universitario Nuestra Señora De Candelaria, Santa Cruz de Tenerife; Ana Belén Escobar Izquierdo, Hospital Virgen De La Salud, Toledo; Javier Estañ Capell, MD, Hospital Clinico Universitario De Valencia, Valencia; Mª Isabel Izquierdo Macián, MD, Hospital Universitari La Fe, Valencia; Mª Mar Montejo Vicente, MD, and Raquel Izquierdo Caballero, MD, Hospital Universitario Río Hortega, Valladolid; Mª Mercedes Martínez, MD, and Aintzane Euba, MD, Hospital de Txagorritxu, Vitoria-Gasteiz; Amaya Rodríguez Serna, MD, and Juan María López de Heredia Goya, MD, Hospital de Cruces, Baracaldo; Alberto Pérez Legorburu, MD, and Ana Gutiérrez Amorós, MD, Hospital Universitario de Basurto, Bilbao; Víctor Manuel Marugán Isabel, MD, and Natalio Hernández González, MD, Hospital Virgen De La Concha - Complejo Asistencial De Zamora, Zamora; Segundo Rite Gracia, MD, Hospital Miguel Servet, Zaragoza; Mª Purificación Ventura Faci, MD, and Mª Pilar Samper Villagrasa, MD, Hospital Clínico Universitario Lozano Blesa, Zaragoza.

SNQ (Swedish Neonatal Quality Register): Zeljka Mustapic, MD, Södra Älvsborgs Sjukhus, Borås; Katarina Strand Brodd, MD, Mälarsjukhuset, Eskilstuna; Andreas Odlind, MD, Falu Lasarett, Falun; Per Friskopp, MD, Gällivare Sjukhus, Gällivare; Sofia Arwehed, MD, Gävle Sjukhus, Gävle; Svetlana Najm, MD, SU/Östra, Göteborg; Anna Kasemo, MD, Länssjukhuset, Halmstad; Karin Nederman, MD, Helsingborgs Lasarett, Helsingborg; Thomas Hägg, MD, Hudiksvalls Sjukhus, Hudiksvall; Fredrik Ingemarsson, MD, Länssjukhuset Ryhov, Jönköping; Henrik Petersson, MD, Länssjukhuset, Kalmar; Ulrik Lindström, MD, Blekingesjukhuset, Karlskrona; Eva Albinsson, MD, Centralsjukhuset, Karlstad; Holger Hövel, MD, Centralsjukhuset, Kristianstad; Carin Widén, MD, Universitetssjukhuset, Linköping; Ingela Heimdahl, MD, Sunderby sjukhus, Luleå; Kristbjorg Sveinsdottir, MD, Skånes Universitetssjukhus, Malmö/Lund; Erik Wejryd, MD, Vrinnevisjukhuset, Norrköping; Johanna Kusima-Löfbom, MD, Skellefteå Lasarett, Skellefteå; Maria Söderberg, MD, Kärnsjukhuset Skaraborg, Skövde; Lars Navér, MD, Karolinska Sjukhuset, Stockholm; Fredrik Stenius, MD, Södersjuhuset, Stockholm; Jens Bäckström, MD, Länssjukhuset, Sundsvall; Peder Helmersson, MD, Norra Älvsborgs Länssjukhus, Trollhättan; Jolanta Wróblewska, MD, Norrlands Universitetssjukhus, Umeå; Erik Normann, MD, Akademiska Barnsjukhuset, Uppsala; Magnus Fredriksson, MD, Visby Lasarett, Visby; Anders Palm, MD, Västerviks Sjukhus, Västervik; Peter Jotorp, MD, Centrallasarettet, Västerås; Bengt Walde, MD, Centrallasarettet, Växjö; Linda Resman, MD, Lasarettet, Ystad; Miriam Pettersson, MD, Universitetssjukhuset, Örebro; Christina Ziegel, MD, Örnsköldsviks Sjukhus, Örnsköldsvik; Kari Arhimaa, MD, Östersunds Sjukhus, Östersund.

SwissNeoNet (Swiss Neonatal Network): Mark Adams, PhD (Network coordinator), University Hospital Zurich; Philipp Meyer, MD, and Rachel Kusche, MD, Cantonal Hospital, Children's Clinic, Aarau; Sven Schulzke, MD, University Children's Hospital, Basel; André Kidszun, MD, University Hospital, Berne; Mathias Gebauer, MD, Children’s Hospital, Biel; Bjarte Rogdo, MD, Children’s Hospital, Chur; Benedikt Huber, MD, Cantonal Hospital, Fribourg; Riccardo E. Pfister, MD, University Hospital (HUG), Geneva; Jean-François Tolsa, MD, and Juliane Schneider, MD, University Hospital (CHUV), Lausanne; Martin Stocker, MD, Children’s Hospital, Lucerne; Ikbel El Faleh, MD, Cantonal Hospital, Neuchatel; Andreas Malzacher, MD, Cantonal Hospital, St. Gallen; André Birkenmaier, MD, Children’s Hospital, St. Gallen; Lukas Hegi, MD, Cantonal Hospital, Winterthur; Vera Bernet, Cantonal Hospital, Zollikerberg; Dirk Bassler, MD, and Vincenzo Cannizzaro, MD, University Hospital (USZ), Zurich; Cornelia Hagmann, MD, University Children’s Hospital, Zurich; Maren Tomaske, MD, Zurich City Hospital, Zurich.

TNN (Tuscany Neonatal Network): Carlo Dani, MD, Careggi University Hospital, Florence, Italy; Marco Moroni, MD, Meyer Children's Hospital, Florence, Italy; Luca Filippi, MD, University Hospital of Pisa, Pisa, Italy; Barbara Tomasini, MD, University Hospital of Siena, Siena, Italy; Elettra Berti, Meyer Children's Hospital, Florence, Italy; Vieri Lastrucci, Meyer Children's Hospital, Florence, Italy.
