# Supplementary material for: Early-Onset Sepsis in Preterm Neonates of 22-28 Weeks’ Gestation: An International Cohort Study
Source: J Pediatr Clin Pract. 2026 May 14;21:200216. doi: 10.1016/j.jpedcp.2026.200216 (PMC13272568; doi:10.1016/j.jpedcp.2026.200216)
Supplement: Data statement [file mmc3.docx]

Data Statement

Data will not be shared, based on the current agreements with individual networks.
